# Confusion, Hallucinations, and Primary Polydipsia: A Rare Presentation of Neurosarcoidosis

**DOI:** 10.7759/cureus.21687

**Published:** 2022-01-28

**Authors:** Hazem Alakhras, Benjamin D Goodman, Markie Zimmer, Sara Aguinaga

**Affiliations:** 1 Internal Medicine, Oakland University William Beaumont School of Medicine, Rochester, USA; 2 Internal Medicine, Michigan State University College of Osteopathic Medicine, East Lansing, USA; 3 Internal Medicine, Beaumont Health System, Royal Oak, USA

**Keywords:** altered mental state, bilateral hilar lymphadenopathy, meningeal enhancement, elevated csf ace, auditory hallucination, visual hallucination, seizure, primary polydipsia, confusion, neurosarcoidosis

## Abstract

Neurosarcoidosis is a rare manifestation of sarcoidosis that can exhibit a variety of neuropsychiatric symptoms and can present independently of pulmonary or other systemic symptoms. This is the case of a 51-year-old African American male who presented with recurrent episodes of auditory and visual hallucinations, confusion, seizures that did not respond to antiepileptics, and recent-onset primary polydipsia. In the emergency department, he did not have meningeal signs, focal neurologic deficits, or a fever. Magnetic resonance imaging (MRI) of the brain demonstrated diffuse meningeal enhancement. The patient underwent a lumbar puncture (LP), with cerebrospinal fluid (CSF) analysis notably revealing an elevated angiotensin-converting enzyme (ACE), an elevated CD4:CD8 ratio, and a negative infectious panel, while computed tomography (CT) imaging showed bilateral hilar lymphadenopathy. He also had an endobronchial ultrasound (EBUS) with biopsy which did not reveal granulomas. Although sarcoidosis requires granulomas for a definite diagnosis, studies and symptoms were consistent with neurosarcoidosis, and this can suggest that the disease was isolated to the central nervous system (CNS). This case highlights the need for further understanding of psychiatric symptoms as a sign of isolated neurosarcoidosis.

## Introduction

Sarcoidosis is a multisystem disorder that is characterized by non-caseating granulomas. Sarcoidosis affects between 59-60 people per 100,000 in the United States of America with an incidence of eight people per 100,000 [1]. It typically presents in African American females with a bimodal distribution including those between the ages of 25-40 and another group between 65-69 [1,2]. Greater than 90% of patients with sarcoidosis present with pulmonary symptoms such as chronic cough and dyspnea, it can also cause mediastinal and hilar lymphadenopathy, but any organ system can be affected including the central nervous system (CNS) and peripheral nervous system (PNS) as in the case of neurosarcoidosis. The pathophysiology is unknown, but is postulated to be due to genetics, environmental mediators, autoimmune, or a result of infectious mediators [3].

Neurosarcoidosis, is an uncommon manifestation of sarcoidosis, presenting in approximately 5-13% of patients with sarcoidosis [4]. Additionally, neurosarcoidosis can be isolated in up to 22% of patients with neurosarcoidosis [5]. The most common symptoms of neurosarcoidosis include peripheral nerve palsies in 52-73% of cases, paresthesias in 43% of cases, and headaches in 37% of cases [6].

Although there is no definitive test for diagnosing sarcoidosis and thus neurosarcoidosis, it is usually based on three major criteria being met. This includes a plausible clinical and radiological presentation, histological evidence of non-caseating granulomas in at least one tissue biopsy, and ruling out other pathologies with similar manifestations such as malignancies or infections [3]. Obtaining histological evidence is often the limiting factor when attempting to secure a definite diagnosis. For sarcoidosis, as pulmonary symptoms are most commonly seen, a biopsy of the lung and hilar and mediastinal lymph nodes using bronchoscopy or endobronchial ultrasound (EBUS) is performed. Neurosarcoidosis often causes inflammation in the brain and meninges, thus, neurologic tissue biopsy is a more invasive endeavor. Therefore, brain and meningeal biopsy is not often performed on living patients, especially when clinical suspicion is high without the tissue confirmation. However, this does mean that meningeal inflammation is likely to show up as enhancement on magnetic resonance imaging (MRI) [6].

Additional laboratory tests can be performed to aid in the diagnosis of neurosarcoidosis. A lumbar puncture can provide valuable evidence. Elevated cerebrospinal fluid (CSF) protein has been noted in 40-83% of cases and lymphocytic pleocytosis in 31-83% of cases [7]. A CD4:CD8 can be obtained from multiple sites including CSF and a ratio greater than five is desired [8]. A CSF angiotensin-converting enzyme (ACE) and IgG index should be obtained as they are usually elevated. A serum interleukin two receptor (sIL-2R) should also be obtained as it has been shown to be more sensitive and specific for sarcoidosis than ACE [9].

Currently, there is no cure for neurosarcoidosis. Many disease courses spontaneously resolve, but can then remit. Standard treatment involves corticosteroids, but benefit from long-term corticosteroids has not been consistently proven. In those patients who may not respond appropriately to corticosteroids, defined as frequent relapses or progression of the disease despite a moderate dose of corticosteroids, immunosuppressants and biologics have been used. These have included azathioprine, cyclophosphamide, hydroxychloroquine, leflunomide, methotrexate, adalimumab and infliximab. Low-dose cranial radiation therapy has been used for refractory cases [3,7]. In this case report, we demonstrate how high clinical suspicion is used to diagnose this patient’s symptoms. We also present a constellation of symptoms which as a whole has not yet been described in English literature.

## Case presentation

Signs and symptoms

A 51-year-old African American male with a reported history of strokes and seizures presented with two days of auditory and visual hallucinations, confusion with schizoaphasia, and lightheadedness with episodes of syncope. He had a similar episode one year prior, which was self-limited and resolved in two days, for which he did not seek medical care. Four months prior, he had a transient episode of left-sided Bell’s palsy, and significant polydipsia and polyuria. Two months prior to presentation to our hospital, he was evaluated at an outside emergency department and was started on levetiracetam 1,000mg BID for an unspecified seizure disorder. Due to a significant decline in mental status, he presented to the emergency department at our tertiary care facility. On admission, he was disoriented to time and place, but oriented to self. He did not have meningeal signs, signs of focal neurologic deficits, and was afebrile and non-toxic appearing. He was inattentive and irritable, demonstrated tangential thought process, pressured speech, impaired recent memory and actively perceived auditory and visual hallucinations. 

Labs and imaging

**Figure 1:**
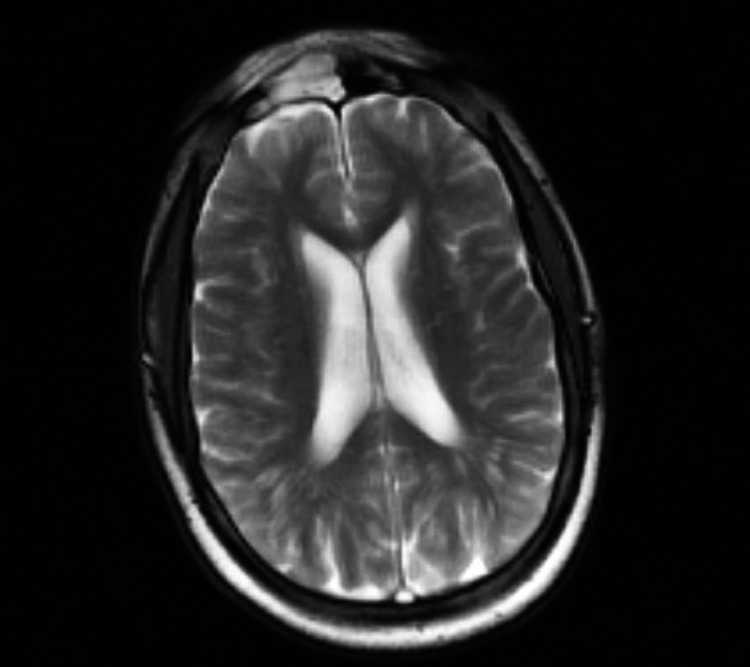
MRI of the brain showing diffuse meningeal enhancement

**Figure 2:**
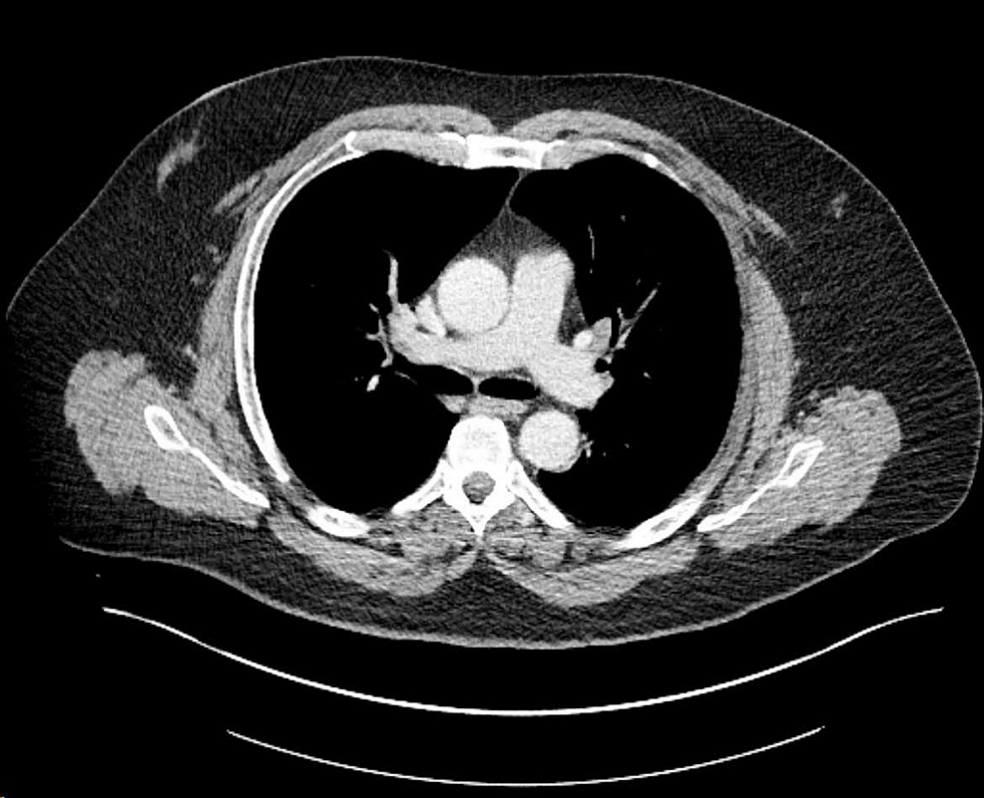
CT of the chest showing bilateral hilar lymphadenopathy

An initial complete blood count showed leukocytosis of 10.5 bil/L, while a comprehensive metabolic panel showed hyponatremia of 131 mmol/L, glucose 93 mg/dL, blood urea nitrogen 9 mg/dL, calcium 9.4 mEq/L. Further investigations showed serum alcohol <10 mg/dL, ammonia 35 umol/L, vitamin B12 406 pg/mL, ceruloplasmin 31 mg/dL, thyroid-stimulating hormone (TSH) 0.78 ulU/mL, and C reactive protein 17.5 mg/dL. Rapid plasma reagin (RPR), Lyme IgG & IgM antibodies, antinuclear antibody (ANA), human immunodeficiency virus 1/2 antigen and antibodies were all negative. Urine studies showed negative drug screens and urine osmolality 67 mOsmol/kg. Throughout hospital admission, urine osmolality increased to 209 mOsmol/kg and his Na corrected to 139 mmol/L, after fluid restriction of 1.5 L per day, markedly decreased from his observed 6-7 L water intake daily. Electroencephalogram (EEG) showed no epileptiform activity. Computed tomography (CT) of the head without contrast showed no intracranial hemorrhage, while MRI of the brain suggested chronic ischemic changes and diffuse meningeal enhancement (Figure 1). This prompted a lumbar puncture (LP) that showed CSF with glucose 35 mg/dL, protein 179 mg/dL, white blood cell (WBC) 18/mcL, oligoclonal bands with IgG 16.3 mg/dL, and ACE 6.0 U/L (normal range: 0.0 - 2.5 U/L). Final CSF culture showed no growth with a negative meningitis/encephalitis infectious panel that included *Neisseria meningitidis*, *Streptococcus pneumoniae*, Enterovirus, and Human Simplex Virus 1 & 2; cytology showed atypical lymphocytes, and flow cytometry showed CD4:CD8 ratio 5.6:1. Chest X-ray was unremarkable, serum ACE 20 U/L, and sIL-2R 700.7 pg/mL. CT of the chest was obtained because of a working diagnosis of possible neurosarcoidosis and showed mediastinal and bilateral hilar lymphadenopathy consistent with sarcoidosis (Figure 2). Quantiferon test was negative and antineutrophil cytoplasmic antibody (ANCA) titers <1:20. A repeat LP was done to more extensively rule out infectious diseases; CSF results were similar with glucose 40 mg/dL, protein 173 mg/dL, WBC 10/mcL, and ACE 5 U/L. Flow cytometry showed CD4:CD8 ratio 7:1 and opening pressure 24.5 cmH₂O. CSF venereal disease research laboratory, fungal antibodies, and acid-fast bacilli culture were all negative. Finally, an EBUS with biopsy of the hilar, paratracheal, and subcarinal lymph nodes was performed, but was limited by low cellularity. Cytology showed no definitive evidence of granulomas or malignant cells, but flow cytometry showed CD4:CD8 ratio 4.3:1.

Management

The patient was given a single dose of haloperidol and lorazepam due to agitation upon admission. He resumed his home medications of aspirin and atorvastatin due to history and imaging consistent with chronic ischemic changes, and also resumed his losartan for hypertension. However, due to his hyponatremia, he was fluid restricted to 1.5 L and his hydrochlorothiazide was discontinued, which corrected sodium levels and his low urine osmolality. His levetiracetam was switched to divalproex for consideration of mood stabilization. He was also given daily thiamine injections and folate supplementation. He was evaluated by Neurology, Psychiatry, Rheumatology, Infectious Diseases, and Pulmonology. His symptoms improved throughout the admission and after a multidisciplinary discussion, initiation of corticosteroids was deferred as the patient’s symptoms spontaneously resolved.

## Discussion

Differential diagnosis

The differential diagnosis of a patient with recurrent episodes of altered mental status is quite broad, but a systematic approach helped guide the diagnosis. A consideration was made to psychogenic causes in light of the visual hallucinations, however, the abnormal MR imaging warranted further investigation into organic causes. Normal blood alcohol levels and urine drug screen, as well as no history of drug use made a toxic encephalopathy unlikely. Normal calcium, blood glucose, TSH, vitamin B12, ammonia, and ceruloplasmin ruled out any metabolic causes such as Wilson’s disease or hepatic encephalopathy. Although the patient was mildly hyponatremic at admission, it was consistent with primary polydipsia and symptoms had not yet improved alongside the correction of sodium. Neurosyphilis, systemic lupus erythematosus (SLE), and CNS vasculitis can present with a wide constellation of neuropsychiatric symptoms, yet a negative RPR, ANA, and ANCA respectively ruled out these possible etiologies. Lyme disease was also considered, though there was no history of recent travel or visible rashes; regardless, Lyme IgG & IgM antibodies were negative. EEG showed no definitive epileptic activity. MRI ruled out acute ischemia, space-occupying lesions, and neurodegenerative changes; however, it did reveal diffuse meningeal enhancement. Although the patient had no fever, headache, myalgia, signs of focal neurologic deficits, or nuchal rigidity, and only had a very mildly elevated WBC, the patient underwent a lumbar puncture to obtain CSF. As the patient did not appear toxic, consideration was given to non-infectious causes of meningitis such as neurosarcoidosis and malignancy. CSF cytology was negative for malignancy, while studies showed decreased glucose, slightly elevated WBC, opening pressure, and protein count which could suggest aseptic meningitis, but a negative infectious meningitis/encephalitis panel and quantiferon test made it less likely and would not explain the hallucinations. In spite of the benign lymph node biopsy findings, the CSF results, in conjunction with an elevated CSF ACE, elevated CD4:8 ratio, and bilateral hilar lymphadenopathy, is highly suggestive of neurosarcoidosis, particularly in the setting of confusion, hallucinations, seizures, primary polydipsia, aseptic meningitis, and episode of Bell’s palsy. 

Analysis

Neurosarcoidosis is a rare form of sarcoidosis, resulting from the non-caseating granulomatous infiltration of CNS and/or PNS. Our patient presented with aseptic meningitis seen in 7-24% of patients, cognitive impairment seen in 2-27%, seizures seen in 2-20%, psychiatric symptoms such as hallucinations seen in 20%, and hypothalamic-pituitary-adrenal axis dysfunction leading to primary polydipsia seen in 2-11% [6,10].

Upon reviewing other case reports of neurosarcoidosis, it is clear that its diagnosis can be challenging due to its unique presentations. One case report described a 36-year-old male with agitation, confusion, and auditory hallucinations who had been misdiagnosed and provided medications that did not fully alleviate the symptoms. He ultimately underwent a brain biopsy which provided a definitive diagnosis of neurosarcoidosis [5]. This highlights the need for a thorough medical investigation in all patients with new-onset psychiatric symptoms. 

More than 90% of patients with sarcoidosis have pulmonary involvement along with granulomatous infiltration of the mediastinal lymph nodes. EBUS is the preferred method of obtaining a biopsy over the traditional bronchoscopy biopsy due to the 80% versus 60% sensitivities in finding non-caseating granulomas, respectively [11]. In our patient, the biopsy from the EBUS did not provide histological evidence for a definitive diagnosis of sarcoidosis. This could be due to the low cellularity from the biopsy, but it could also indicate that our patient presented with isolated neurosarcoidosis. 

A CD4:8 ratio can be used to aid in the diagnosis of sarcoidosis as CD4+ T cells are thought to play an integral role in the disease pathology [8]. We were able to obtain CD4:8 ratios from both CSF samples yielding 5.6:1 after the first LP and 7:1 after the second LP. An EBUS biopsy also yielded a CD4:CD8 ratio of 4.3. It has been suggested that a ratio greater than or equal to five can aid in the diagnosis of neurosarcoidosis. This was expanded on by a recent study that evaluated CSF results for patients with neurosarcoidosis and noted that, on average, the CSF CD4:CD8 ratio was 4.2, adding to our suspicion of neurosarcoidosis. Additionally, the authors observed that when there was also an elevated CSF lymphocyte count greater than the upper limit of normal, as was with our patient, the positive predictive value, negative predictive value, and specificity was 57%, 88%, and 95%, respectively [8]. 

ACE is derived from epithelioid cells of active macrophages and can be used to monitor disease severity and the granulomas in the individual’s body. Of course, elevated ACE can be seen in other granulomatous diseases along with non-granulomatous diseases [12]. Our patient’s CSF ACE was elevated, while his serum ACE was within normal limits. CSF ACE is useful in the diagnosis of neurosarcoidosis with a sensitivity of 55% and a specificity of 94% [13]. While serum ACE has a sensitivity of 62% and a specificity of 76% in the diagnosis of sarcoidosis [9]. However, serum ACE is elevated only up to 35% of the time in neurosarcoidosis, and is more likely to be normal when isolated [5].

## Conclusions

Based on the patient’s symptoms and radiological and laboratory findings, we believe that a diagnosis of neurosarcoidosis is most probable. Although there are a few laboratory findings that did not meet the necessary cutoffs, it is important to note that no one test attains a definitive diagnosis or has a 100% sensitivity or specificity for sarcoidosis, particularly if neurosarcoidosis is isolated. As mentioned previously, histological evidence is necessary to provide a definitive diagnosis of any form of sarcoidosis. Therefore, a biopsy of the brain in the areas that are enhanced on MRI would be the diagnostic step. However, this was deferred given patient improvement and the fact that it would not change management at the current time. 

The patient was discharged with a presumptive diagnosis of neurosarcoidosis and was counseled on its manifestations. He was instructed to continue divalproex, as well as fluid restriction. At the time of this writing, he has had no recurrent episodes or new symptoms since his recovery. This case highlights the importance of thorough medical evaluation of psychiatric symptoms. It also serves as a reminder that neurosarcoidosis can present in isolation from other systemic manifestations of sarcoidosis. Thus, when clinical suspicion is high, an LP may be necessary.
